# Rare Presentation of HHV‐7 Meningitis in a Nonimmunocompromised Adult: A Case Report and Literature Review

**DOI:** 10.1155/crdi/8512795

**Published:** 2026-10-15

**Authors:** Ismail Al Hajj, Sara Farhat, Ghenwa El Dakdouki

**Affiliations:** ^1^ Internal Medicine Faculty of Medicine Beirut Arab University Beirut Lebanon bau.edu.lb; ^2^ Internal Medicine Faculty of Medicine Lebanese University Beirut Lebanon ul.edu.lb; ^3^ Internal Medicine Infectious Diseases Hammoud Hospital University Medical Center Saida Lebanon hammoudhospital.com

**Keywords:** HHV-7, immunocompetent adult, meningitis

## Abstract

**Background:**

Human herpesvirus‐7 (HHV‐7) is a member of the herpesvirus family that is commonly asymptomatic during infection, but it can lead to the development of exanthema subitum. Although HHV‐7 primarily affects children and establishes latency in the body, reactivation of HHV‐7 can occur in individuals with compromised immune systems and those using immunosuppressive medications, resulting in diverse clinical manifestations. However, the acute HHV‐7 presentation in immunocompetent adults is uncommon.

**Case Presentation:**

Here, we report a rare presentation of HHV‐7 meningitis in an immunocompetent adult.

**Conclusion:**

This case report should raise concern about the atypical presentation of HHV‐7 infection in an immunocompetent adult host, suggesting the urge to expand investigation on the pathogenicity of HHV‐7 for better understanding of the mechanism of infection, early clinical intervention, and avoidance of further manifestations and death.

## 1. Introduction

Human herpesvirus‐7 (HHV‐7), a double‐stranded DNA lymphotropic virus belonging to the Roseolovirus genus among Herpesviridae [1], is a ubiquitous herpesvirus with seropositivity among adults reaching more than 95% [2]. HHV‐6A, HHV‐6B, and HHV‐7 comprise the Roseolovirus genus of the subfamily β‐Herpesvirinae [3]. Once the primary infection has occurred, HHV‐7 maintains latency in CD4 T cells and is continuously shed in saliva thereafter [4, 5]. Some patients can develop exanthema subitum, which is seen more commonly with HHV‐6 infection [5]. About 50% of hematopoietic stem cells and 20% of solid‐organ transplant recipients reactivate HHV‐7 as indicated by viral DNA in the peripheral blood [6]. HHV‐7 reactivation and related diseases mostly occur in immunocompromised patients, as reported in the literature [7].

Meningitis is defined as inflammation of the meninges and is classified as aseptic or bacterial. Aseptic meningitis is more common and is usually due to viral pathogens, with Enterovirus being the most common cause in nonimmunocompromised patients [8].

HHV‐7 reactivation causing CNS manifestations is fairly reported in the literature in patients with an immunocompromised state but not in immunocompetent patients. However, few case reports described HHV‐7 as a cause of meningoencephalitis in immunocompetent patients. In this case report, we will discuss a case of meningitis in an immunocompetent adult, a rare manifestation of HHV‐7. A comprehensive review of HHV‐7‐associated CNS infections has previously been published [9]. To provide an updated overview of the available evidence, we reviewed cases reported after the publication of that review, focusing on newly described clinical presentations, diagnostic approaches, therapeutic strategies, and outcomes. A literature search was conducted in PubMed from January 2017 to June 2026 to identify recent additional cases of HHV‐7‐associated CNS infections. Search terms included “human herpesvirus 7,” “HHV‐7,” “encephalitis,” “meningoencephalitis,” and “central nervous system infection’.”

## 2. Case Presentation

A 31‐year‐old, previously healthy male with a nonsignificant past medical, surgical, family, or psychosocial history, presented to our hospital after 3 weeks of experiencing headache and fever that were not relieved by over‐the‐counter paracetamol. The headache was occipital, bilateral, alleviated with neck movement, and persistent throughout the day with no radiation. He also complained of associated photophobia, nausea, and vomiting. There was no rash before or during the onset of symptoms. His family denied any alteration in the level of concentration or mental status.

In the emergency department, the patient was conscious and oriented to date, time, and place. He had a fever of 39.2°C, a normal blood pressure of 120/70 mm·Hg, a pulse rate of 110 beats per minute, and an oxygen saturation level of 97% on room air. The patient also exhibited neck stiffness, known as nuchal rigidity. Kerning and Brudzinski’s signs were both positive. On review of systems, he had regular S1S2 with no murmur, good bilateral air entry with no adventitious sounds, soft and nontender abdomen with no organomegaly, and intact cranial nerves function from I to XII.

Blood tests for blood glucose and electrolytes, as well as kidney function and urine analysis, all came back normal. Brain computed tomography (CT) and magnetic resonance imaging (MRI) revealed no acute intracranial abnormalities or focal lesions, and no radiologic evidence of encephalitis. Cerebrospinal fluid (CSF) analysis revealed 236 cells per microliter with a 74% lymphocytic predominance, normal glucose of 57 mg/dL, and an elevated protein level of 119.2 mg/dL, findings consistent with aseptic meningitis. Testing for human immunodeficiency virus (HIV) and brucella was negative; other laboratory test results are shown in Table 1 (Table 1).

**TABLE 1:** Laboratory data on admission.

Variable	Reference range of adults	On admission
Hematocrit (%)	40–54	41.8
Hemoglobin (g/dL)	13–18	14.0
White‐cell count (perµl)	4–11	7.04
Neutrophils (%)	40–65	79.3
Lymphocytes (%)	25–40	16.5
Monocytes (%)	2–8.0	3.4
Eosinophils (%)	0–4	0.4
Platelet count (10^3/μL)	150–400	251
Alkaline phosphatase (IU/L)	30–120	48
Alanine aminotransferase (U/L)	< 45	35
Aspartate transaminase (U/L)	< 35	56
Total bilirubin (mg/dL)	0.3–1.2	0.6
Direct bilirubin (mg/dL)	0–0.5	0.2
Albumin (g/dL)	3.5–5.2	4.4
C‐reactive protein (mg/L)	< 5	38.6
CSF results:		

WBC/cu.mm		236
RBC/cu.mm		0
Lymphocytes (%)		74
Neutrophils (%)		26
CSF glucose (mg/dL)	40–70	57
CSF protein (mg/dL)	15–45	119.2

While awaiting the results of the meningitis multiplex PCR, empirical acyclovir (750‐mg IVD Q8) and ceftriaxone (2g IVD Q12) were started for suspected meningitis. Steroids were not started initially, but they were introduced 3 days after presentation (dexamethasone 8‐mg IVD once daily). The patient kept getting high fevers despite antipyretic usage (paracetamol 1 g Q6 hours) (Figure 1). Meningitis multiplex PCR (using the BioFire FilmArray Meningitis/Encephalitis Panel) result on the seventh day of hospitalization detected the presence of HHV‐7 alone. By this result, a diagnosis of HHV‐7 meningitis was confirmed, and ganciclovir treatment was started at 5 mg·per kg twice daily. Within 48 h of ganciclovir induction, the patient showed significant clinical improvement in fever and headache, as well as in the level of concentration. The patient received ganciclovir over a total of 5 days and was discharged home afebrile and asymptomatic.

**FIGURE 1:**
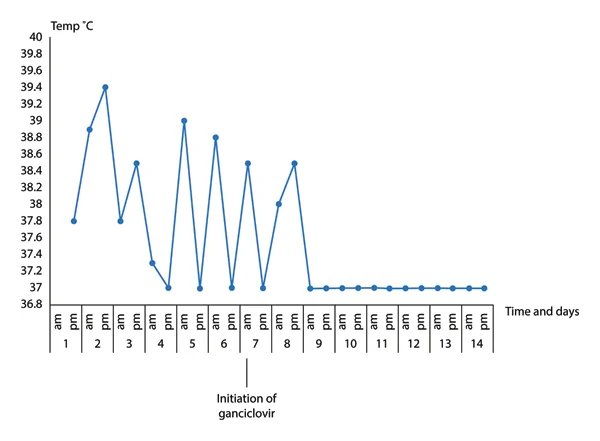
Temperature charting during the hospital stay.

## 3. Discussion

It is important to differentiate between encephalitis and meningitis. In meningitis, the patients might experience fever, headache, and nuchal rigidity, but their brain function is not affected. In encephalitis, however, the state of consciousness can be altered. The patient in the presented case had headache, fever, and neck stiffness, with no altered mental status, which meets the criteria of meningitis. The results of CT and MRI in this patient demonstrated no abnormal radiographic findings, and the results were inconclusive.

HHV‐7 infection is usually asymptomatic and is similar in clinical presentation to HHV‐6, as shown in several studies. Although it can present with meningitis or encephalitis in immunocompromised and stem cell transplant patients, it is even rarer to cause meningoencephalitis in immunocompetent adults (Table 2).

**TABLE 2:** Summary of reported cases.

Case number	Clinical diagnosis	Age	Gender	Presentation	Microbiological HHV‐7 diagnosis	CSF	Primary vs. secondary infection	Treatment	Reference
1	Limbic Encephalitis	17	Male	Status Epilepticus, headache	HHV‐7 DNA in CSF (mNGS)	—	—	Acyclovir	Lynch et al. [10]
2	Meningitis	37	Female	Radiating back pain, numbness in limbs	HHV‐7 in CSF Panel PCR	—	—	Dexamethasone, ceftriaxone	Nguyen et al. [11]
3	Meningitis	17	Female	Headache, neck stiffness, fever	HHV‐7 DNA in CSF	WBC 30 count/mL (PMN = 70% and lymphocytes = 30%), RBC 80 count/mL, glucose 52 mg/dL, protein 35.5 mg/dL	—	Acyclovir	Farahani et al., 2023 [12]
4	Encephalitis	26	Female	Fever, mental and behavioral disorders, coma, and intermittent convulsions	HHV‐7 DNA in CSF (mNGS)	Leucocytes: 1, glucose 0.504 g/L, protein 0.341 g/L,	—	Acyclovir and ganciclovir	Li et al., 2022 [13]
5	Encephalitis and inflammatory demyelinating disease Overlapping syndrome	28	Male	Headache, dizziness, fever, optic neuritis, and epileptic seizures	HHV‐7 DNA in CSF (mNGS and PCR)	WBCs: 610/mm^3^, protein 1.28 g/L, normal glucose	P	Ganciclovir	Li et al., 2022 [14]
6	Encephalitis	20	Male	Partial seizures with secondary generalization, headache, and asthenia	HHV‐7 DNA in CSF by Multiplex Nested PCR	132 leucocytes/μL (lymphocytes + monocytes) and 100 red blood cells/μL, 42 mg/dL of protein, and 60 mg/dL of glucose (blood glucose, 98 mg/dL)	—	No treatment	Distefano et al., 2019 [15]
7	Limbic encephalitis	35	Male	General fatigue, fever, headache, vomiting, consciousness disturbance, and seizures	HHV‐7 DNA in CSF	WBC: (35/mm^3^); lymphocytes (94%), protein level (76.5 mg/dL), glucose level (113 mg/dL	R	Methylprednisolone, intravenous immunoglobulins, and acyclovir.	Aburakawa et al., 2017 [16]
8	Encephalitis	36	Male	Headache, diplopia, nausea, vertigo, asthenia, and fever	HHV‐7 DNA in CSF	WBC: 128 cells/mm3; glucose (66 mg/dL); proteins (59 mg/dL)	R	Acyclovir and dexamethasone	Riva et al., 2017 [17]

*Note:* Nested PCR performed on CSF sample was also positive for cytomegalovirus (CMV); IFA, indirect immunofluorescence assay; P: primoinfection; R: reactivation, —: not mentioned/unknown. NB: The immune status of all patients mentioned in the table above is immunocompetent.

Abbreviation: PE, postinfectious encephalomyelitis.

Reported cases of HHV‐7 CNS infections in immunocompetent adults (Table 2), defined by a positive PCR on CSF, showed that the infection may manifest with encephalitis alone, or can be associated with other neurological symptoms including headache, fever, neck stiffness, nausea and vomiting, dizziness, vertigo, diplopia, sensory disturbances such as limb numbness, altered consciousness, behavioral or mental changes, seizures, and, in severe cases, coma [18–28].

In contrast to similar reported cases, our patient did not exhibit any notable symptoms aside from photophobia, fever, headache, and neck stiffness. Specifically, there was no rash, paralysis, convulsions, seizures, focal neurological deficit, spinal or cranial nerve involvement, or any other organ manifestations.

In some cases, multiple organisms, including HHV‐7, might be detected in the CSF of a patient with suspected meningitis. Sgarabotto et al., described a case of meningoencephalitis in a 21‐year‐old man in whom standard DNA PCR on CSF identified the presence of 3 pathogens (HHV‐6, HHV‐7, and *Mycoplasma pneumoniae*) [29]. In contrast, in our case, HHV‐7 was the only pathogen identified on PCR of the CSF.

HHV‐6 DNA may be detected in the CSF in association with other inflammatory or neurological disorders, without necessarily indicating active CNS infection [29, 30]. HHV‐6 might also be present in the chromosomally integrated form (ciHHV‐6), which is present in approximately 1% of individuals and results in persistent detection of HHV‐6 DNA in multiple body fluids [31, 32].

Similarly, HHV‐7 can integrate into human telomeres, as demonstrated experimentally, and possible chromosomal and germline integration has also been reported [33, 34]. However, unlike ciHHV‐6, germline‐integrated HHV‐7 appears to be uncommon, and its prevalence and clinical significance remain uncertain. Importantly, the detection of HHV‐7 DNA in CSF does not necessarily establish an etiologic association with the underlying neurological disease [35]. Nevertheless, Corral et al. found that HHV‐7 DNA detection in adult CSF had high specificity and suggested that HHV‐7 may represent a causative pathogen in a subset of patients with compatible CNS infection and no alternative etiology [36]. Therefore, HHV‐7 should be considered in, otherwise, unexplained CNS infections, with PCR results interpreted in conjunction with the clinical presentation and CSF findings.

Ganciclovir, acyclovir, foscarnet, and intravenous immunoglobulin have been used in reported cases of HHV‐7 encephalitis. However, clinical improvement has also been reported in some cases without antiviral therapy [13]. In the report by Farahani et al. [37], they described a case of a 17‐year‐old adolescent girl that had similar symptoms in comparison to our case and also HHV‐7 was detected on CSF; however, symptoms were preceded by maculopapular rash of 1‐day duration and treatment with Acyclovir was successful in retaining the patient symptom free and then discharged with no complication.

In our case, the patient showed no clinical improvement despite a six‐day course of empirical acyclovir therapy and three days of dexamethasone. When HHV‐7 DNA was detected via PCR on CSF, acyclovir was discontinued, and ganciclovir was initiated. Within three days of starting ganciclovir, the patient had an excellent clinical response where the headache subsided and temperature returned to normal (Figure 1), so we maintained this therapy.

In the study by Fule Robles et al., multiple reports were cited indicating that the efficacy of foscarnet and ganciclovir in treating HHV6 or HHV‐7 infections remains a matter of debate [7]. Thus, for patients with severe HHV‐6 or HHV‐7 infections, it may still be advisable to consider a trial of foscarnet and/or ganciclovir. However, prospective studies evaluating treatment strategies for HHV‐7 central nervous system (CNS) infections remain limited.

Upon reviewing the literature (Table 2), we noted that there is male‐to‐female predominance concerning the presentation of HHV‐7 in immunocompetent adults, which might be incidental or of certain significance and warrants further investigations.

## 4. Conclusion

In conclusion, this report describes a rare case of HHV‐7 meningitis in an adult immunocompetent patient with no focal neurological deficit or other organ manifestation. Although HHV‐7 CNS infection is typically self‐limited in healthy individuals, immunocompromised patients may require antiviral therapy. Ganciclovir and foscarnet are the primary therapeutic options for HHV‐7 CSF infection, but their efficacy remains a matter of debate. In our case, the patient showed an excellent clinical response to ganciclovir therapy after failing to respond to acyclovir and dexamethasone. Further studies are needed to better understand the optimal management of HHV‐7 CNS infection in both immunocompetent and immunocompromised patients.

### 4.1. Strengths and Limitations

The main strengths of this study are the rarity and clinical relevance of HHV‐7 meningitis in an immunocompetent adult, detection of HHV‐7 as the sole pathogen by multiplex CSF PCR, documented clinical response to ganciclovir, and an updated literature review. Limitations include the single‐case design, which limits generalizability and prevents establishing causality or treatment efficacy, and the lack of quantitative HHV‐7 viral load or repeat CSF testing to confirm active CNS infection.

## Author Contributions

All authors participated in the patient’s clinical care, contributed to the conception and preparation of the manuscript, and critically reviewed the content.

## Funding

This study was conducted without financial support from any funding agency in the public, commercial, or not‐for‐profit sectors.

## Disclosure

All authors have approved the final version for publication.

## Ethics Statement

In accordance with institutional policy, formal ethical approval was not required for this single‐patient case report. Written informed consent for participation and publication was obtained from the patient.

## Consent

The patient provided written informed consent for the publication of this case report and any accompanying clinical information.

## Conflicts of Interest

The authors declare no conflicts of interest.

## Data Availability

All relevant data are included within the article. Further information is available from the corresponding author upon reasonable request.
